# Edinburgh Infirmary

**Published:** 1831-07

**Authors:** 


### EDINBURGH INFIRMARY.

### *Remarkable Case of Subacute Peritonitis; Death.**

Agnes Conolly, ætat. twenty, a servant, admitted March 26th. Headach and pain of back; vertigo upon sitting up; slight pain under the left mamma; cough, stated to be of some months standing, without recent aggravation; frequent chills, alternating with flushings; skin soft; pulse ninety-six, full; tongue white, and furred in centre; thirst and anorexia: countenance natural; bowels reported open. Four days ago had a rigor, followed by present symptoms. Was bled and had an emetic yesterday. Was lately visiting some persons labouring under fever. Sumat Infusi Cathartici, ℥iv.

27th. Several dark-coloured stools; countenance nearly natural; headach, and pain of back. Pulse 112, moderately full and firm; respiration thirty-eight; complains much of pain under the left mamma, not aggravated by pressure, and slightly by full inspiration; the râle souscrepitant is distinct there; natural respiration over the rest of the chest; pretty frequent cough, exciting little pain; expectoration mucous, frothy without tinge. Tongue rather florid, slightly furred. No cough from full inspiration. Fiat V.S. Sumat. Solut. Tart. Antim. ℥ss. ad nauseam sustinendam. Lateri dolenti applic. vesicator. Rep. Haust. Cathartic.

* Medical Gazette.

28th. Syncope and sweating after ℥xiv. of blood had been removed, but no change of symptoms; bleeding repeated in the evening to ℥xii., causing much nausea; blood natural; headach occasionally returns; pain of side and back easier. Pulse 112, firm, though she still complains of nausea; respirations twenty-eight, heaving. Tongue partially furred; yellow frequent stools. Blister rose well; slept well, and was free from pain in the night. Had an opiate draught. Occasional vomiting. Sputa muco-purulent. Says she has dyspnœa occasionally, when otherwise in perfect health, and that she is subject to palpitations. Contin. Solut. Antimon. et Haust. h. s.

29th. Much nausea, no vomiting; giddiness; pain of side slight, and only occasional, not excited by coughing or full inspiration. Pulse 116, moderately firm. Tongue, lips, and teeth, encrusted. Skin of moderate warmth; slight mucous and sonorous râles in left side of chest; expectoration free; sputa as before; less cough; respirations twenty-eight. Omit. Solut. Antim. Contin. Haust. h. s. et Infus. Cathartic.

30th, eighth of disease. Restless and incoherent in the night. Cathartic draught taken at five this morning; shortly after, one scanty stool, containing some blood; three or four stools at considerable intervals, containing more or less blood, and within the last twenty minutes the discharge has been profuse, with great languor, pale countenance, and feeble pulse; skin of natural temperature. Injic. quamprimum Enema ex aquâ frigidâ, ℥iv. et Tinct. Opii, ʒiss. Post horam unam hæmorrhagiâ perstante injic. Enema ex Decoct. Quercus ℥iv., Tr. Opii, ʒj. Acetat. Plumbi, gr. iv. Sumat subinde, gtt. x. Acid. Sulph. Dilut. ex aquâ frigidâ. Vinum pro re natâ.

31st. Injection almost immediately returned, with many large clots of blood; repeated immediately, and partly retained. Bark injection not required. No stool; has had ℥v. of wine; slept in the night, but talked much. Pulse 120, much more distinct and firmer; much thirst. Tongue furred, white, soft; lips pale, and partially covered with black crusts. Stat. injic. Enema Domest. Sum. indies Vini Rubri, ℥vj. Omit. Acid. Sulph. Dil. To have a pound of lemonade.

April 1st. Enema returned, deeply tinged with blood, but without fæces; shortly after, at short intervals, had repeated discharges of small quantities of blood. Bark injection then given, and retained till morning; since which four stools, very dark, liquid, partly feculent and fetid, containing a few clots of blood. Pulse 112, moderately full, of moderate strength, easily compressed. Countenance very pale and depressed. Slept, but talked much during the night. Skin soft, of moderate heat. Tongue furred, white; respirations heaving, less frequent. Wine relished: contin. Vinum. To have ℥iv. of calf's-foot jelly, containing ℥ij. of wine and sugar. Imperial for drink, without sugar.

2d. Jelly relished. Several stools, pretty feculent, of mixed colour, with some slime, and several clots of blood. Dyspnœa in the night, to which she is subject; subsided towards morning; mouth rather cleaner; pulse 108, of moderate strength. Contin. ut heri.

3d. Four stools; the two last more feculent, and without blood. Less thirst; slept well, and is still drowsy. Pulse 120, of moderate strength; thinks herself better; mouth parched; dislikes the jelly and wine. Omit. Vinum Rub. et hab. vice vini albi Hispan. ℥viij. Omit the jelly. To have a pound of very strong beef-tea daily.

4th, thirteenth of disease. Several stools, some of them black, others more natural, though slimy, and containing no blood. Pulse 140, pretty full and distinct, easily compressed. Wound made in bleeding has not healed, discharges pus; the vein is felt hard for an inch above, and more than twice as much below it, in the natural situation, accompanied with surrounding hardness and much pain on pressure. Considerable pain in the hypogastrium, aggravated by pressure, but without distention or hardness; some difficulty in voiding urine. Foveatur Hypogast. Brachio dolent. applic. Hirud. xii. Omit. Vinum. Contin. Jusculum Bovinum.

5th. Six leeches fastened and bled well; in the afternoon, pulse 160, small and indistinct; complained of faintness; no complaint of arm; pretty frequent stools; had an opiate enema, and the wine was renewed. Pulse continued to sink; brandy was given; vomiting occurred at midnight, and she died at five this morning, without preceding drowsiness or delirium.

*Sectio cadaveris, thirty-six hours after death.* The coats of that part of the vein which had felt hard during life, were considerably thickened and firm; and for more than half an inch above and below the puncture there was a distinct coating of lymph upon its inner surface; only some minute spots on the other parts; no pus within the tube of the vein; a clot of blood at the lower part, where the sound and diseased portions joined. The vein above and below the hardened portion, and the collateral veins, were healthy. The cellular and adipose substance around the diseased portion of vein were dense and firm.

Upon opening the abdomen, large and numerous patches of soft lymph were scattered over most of its viscera, but chiefly upon the small intestines; and there were several ounces of serum, turbid from flakes of lymph, in the depending portions. The intestines were collapsed, containing no flatus, and scarcely any fæces; numerous florid and arborescent vascular bands and patches on their peritoneal coat, with a corresponding appearance in the peritoneal lining of the abdominal parietes. A small perforation about twice the size of a pin's head, was found in the lower part of the ileum, about twelve inches from where it enters the caput cæci; and in the corresponding part of the inner surface there was an irregular ulceration, without surrounding thickening or vascularity, of the size of a half-crown, part of the bottom of which was formed by the peritoneum alone. Five or six smaller ulcers lower in the ileum, some of them extending through the mucous coat, others down to the peritoneal. The mucous coat of the great intestines appeared healthy, as far as could be judged from a hurried examination. No fæces, or fæculent odour, in the cavity of the abdomen. Liver large, with hypertrophy of the grey matter. Lungs voluminous, considerably emphysematous, and crepitating every where.

Though this case illustrates several pathological facts of considerable importance, yet it appears to me, that by far its most interesting feature is the occurrence of such extensive and rapid peritonitis, with such trifling accompanying symptoms. No doubt extensive effusions of lymph and pus do occasionally take place into the cavity of the abdomen in a very insidious manner; but these, as far as I know, differ in some essential points from the present. They have either taken place in a slow and gradual manner, forming examples of chronic peritonitis, or they have been accompanied with cerebral derangement, blunting sensation. Since morbid anatomy has become so much cultivated, extensive traces of inflammation occurring when the patient had been much debilitated by previous disease, are often discovered after death, the existence of which was by no means indicated by proportionate symptoms, or where they have been so slight as to have been entirely overlooked. In peritonitis from perforation of the intestines, even in the most exhausted condition of the body, when the sensation is unaffected, its occurrence is most generally marked by acute pain which may, however, be of short duration, soon followed by vomiting and tympanitic distention. In some cases the pain has been so excruciating, and attended with such depressing effects upon the heart and arteries, as to have proved fatal within twelve, or even six hours. In the present case, with the exception of some delirium during one night only, the mind was always perfectly coherent; little, if any, of the confusion of thought usual in fever was observable, and there was no drowsiness. It will be perceived from the reports, that the pain of abdomen is only mentioned once, and is the very last thing in that report, shewing how little she complained of it. In the afternoon she denied all pain of abdomen. This, however, is less wonderful, for by this time the pulse had become very feeble; and it is nothing unusual, in cases of inflammation, for the pain to abate almost entirely for several hours before death. The remarkable thing is, that the pain should have been so slight at the very commencement. How few of the usual symptoms of peritonitis were present, may also be seen from the reports. There was no distention of abdomen; instead of constipation, the stools were frequent, requiring the exhibition of an opiate enema; and there was no vomiting, except a very few hours before death; and this occurs so frequently about this period in various diseases, that little can be trusted to it. There was certainly a decided change for the worse in the state of the pulse, and the appearance of the patient, dependent, no doubt, principally upon the peritonitis; but the appearance of the phlebitis at the same time seemed to account for this change. If from mere pain of abdomen alone, continuing only for a short time, while the presence of a local affection apparently accounted for all the other symptoms which may have appeared about the same time, we were always to suppose that this was a case of peritonitis, from perforation of the intestines, I am afraid that scarcely a day would pass over our heads without committing serious errors.

---

### CRITICAL ANALYSES.

